# The protective effects of polysaccharide extract from Xin-Ji-Er-Kang formula on Ang II-induced HUVECs injury, *L*-NAME-induced hypertension and cardiovascular remodeling in mice

**DOI:** 10.1186/s12906-019-2539-z

**Published:** 2019-06-13

**Authors:** Ling Ding, Pan Cheng, Li Wang, Juan Hu, Yong-xue Zhang, Guo-wei Cai, Guang-yao Huang, Shan Gao

**Affiliations:** 1 Department of Pharmacology, Basic Medical College, Anhui Medical University, Hefei, China; 2 Cancer Hospital, Chinese Academy of Sciences, Hefei, 230032 China

**Keywords:** Hypertension, Cardiac remodeling, Endothelial dysfunction, Oxidative stress, Inflammation

## Abstract

**Background:**

Xin-Ji-Er-Kang (XJEK) is a Chinese herbal formula, which has been reported to exert effective protection against cardiovascular diseases, including hypertension and myocarditis.

**Methods:**

Cultured human umbilical vascular endothelial cells (HUVECs) were treated with angiotensin II (Ang II) and different concentrations of aqueous layer extracts (AqE). Subsequently nitric oxide (NO) and endothelial nitric oxide synthase (eNOS) expression levels were detected. In addition, fifty Kunming mice were randomized into control, Nω-nitro-*L*-arginine methyl ester (*L*-NAME), *L*-NAME+AqE, *L*-NAME+XJEK and *L*-NAME+fosinopril treatment groups. Following 8 weeks of treatment, the cardiac hemodynamic index was measured, relaxation of the aorta was examined and pathological changes were observed. Colorimetric analysis and enzyme linked immunosorbent assay (ELISA) were applied to determine the relevant indicators in plasma and cardiac tissues.

**Results:**

The in vitro study results demonstrated that AqE could preserve endothelial function (NO, 21.05 ± 2.03 vs. 8.64 ± 0.59; eNOS, 1.08 ± 0.17 vs.0.73 ± 0.06). In addition, the in vivo results demonstrated that compared with the control group, treatment with AqE could enhance a high hemodynamic state (left ventricular systolic pressure, 116.76 ± 9.96 vs.114.5 ± 15.16), improve endothelial function (NO, 7.98 ± 9.64 vs. 1.66 ± 3.11; eNOS, 19.78 ± 3.18 vs.19.38 ± 3.85), suppress oxidative stress (OS) (superoxide dismutase, 178.17 ± 13.78 vs. 159.38 ± 18.86; malondialdehyde, 0.77 ± 0.13 vs.1.25 ± 0.36) and reverse cardiovascular remodeling.

**Conclusion:**

Polysaccharide from XJEK exerts protective effects against Ang II-induced injury in HUVECs and *L*-NAME-induced hypertension in mice and the underlying mechanism may be attributed to improving endothelial dysfunction, OS and the inflammation status in mice.

## Background

Hypertension is a significant health threat in the USA due to its mortality rate and treatment costs. Approximately 31% of Americans have been diagnosed with hypertension [1]. In addition, it is a major risk factor for fatal diseases, including stroke, atherosclerosis and congestive heart failure. Evidently, effective control of blood pressure reduces the morbidity and mortality rates of these diseases [2, 3]. Recently, the curative effects of traditional Chinese medicine (TCM) have received growing attention both in China and abroad. The therapeutic effect of a typical Chinese herbal formula (Table 1) Xin-Ji-Er-Kang (XJEK) in hypertension-induced coronary heart disease, toxic myocarditis and virus myocarditis has been confirmed in both clinical and basic studies [4, 5]. It has been demonstrated that the underlying mechanism of XJEK may involve decreasing oxidative stress (OS) and improving endothelium function [6, 7].

**Table 1:** Recipe of XJEK formulation

Component	Voucher number	Specimens used	Part rate (%)
*Panax ginseng C.A. Mey.*	NJUTCM-20110530	Root	11.71
*Polygonatumodoratum (Mill.) Druce*	NJUTCM-20110531	Rhizome	7.03
*Panax pseudoginseng var. Notoginseng (Burkill) G. Hoo& C.L. Tseng*	NJUTCM-20110532	Root	3.09
*Allium macrostemon Bunge*	NJUTCM-20110533	Ramulus	7.80
*Angelica sinensis (Oliv.) Diels*	NJUTCM-20110534	Root	7.80
*Ophiopogonjaponicus (Thunb.) Ker Gawl.*	NJUTCM-20110535	Root	7.80
*Schisandrachinensis (Turcz.) Baill.*	NJUTCM-20110536	Fruit	3.93
*Salvia miltiorrhiza F. alba C.Y. Wu & H.W. Li*	NJUTCM-20110537	Root	7.80
*SophoraflavescensAiton*	NJUTCM-20110538	Root	7.80
*Glycyrrhizaacanthocarpa (Lindl.) J.M. Black*	NJUTCM-20110539	Rhizome	7.80
*Astragalusmongholicus Bunge*	NJUTCM-20110540	Root	11.69
*EpimediumacuminatumFranch.*	NJUTCM-20110541	Aerial part	7.80
*Trichosanthes obtusiloba C.Y. Wu*	NJUTCM-20110542	Seed	7.80
*Dryobalanopsaromatica C.F. Gaertn.*	NJUTCM-20110543	Resin	0.15

*Cited from our previous report (Guo* et al.*, Journal of Ethnopharmacology, 2014, 155, 1227–1235)*

Furthermore, an increasing amount of evidence has revealed various beneficial medicinal effects of polysaccharides in TCM, including antitumor [8], antivirus [9, 10] and anti-oxidative effects, and regulation of the immune system and cytokine secretion. Notably, several main components of XJEK, such as *Polygonatum odoratum* (Mill.) Druce, *Astragalusmongholicus* Bunge and *Ophiopogon japonicus* (Thunb.) Ker Gawl exert protective effects in cardiovascular diseases [11–14]. In our previous study, it was implied that polysaccharides from XJEK decoction exhibited an anti-cardiac remodeling effect in an isoprinosine-treated mouse model [15]. Recent research demonstrated that XJEK formula has a protective effect against Nω-nitro-*L*-arginine methyl ester (*L*-NAME)-induced hypertension and angiotensin II (Ang II)-induced Human umbilical vascular endothelial cells (HUVECs) injury [16, 17], but the effect of polysaccharides from XJEK decoction on these two models has not been elucidated.

Chronic administration of nitric oxide (NO) synthase inhibitors, including *L*-NAME, can lead to severe chronic hypertension, which is a method commonly used for the establishment of animal models with pharmacological hypertension. In addition, administration of NO synthase inhibitors has been associated with decreased NO bioactivity and endothelial dysfunction (ED). Furthermore, as suggested by recent studies, NO deficiency is directly associated with the generation of inflammatory cytokines, including tumor necrosis factor-α [18, 19]. Therefore, the anti-hypertensive effects of polysaccharide extract from XJEK were investigated in the present study in *L*-NAME-induced hypertensive mice, and the underlying molecular mechanism of cardiovascular protection was assessed by examining ED, anti-oxidative stress and anti-inflammatory activities.

## Materials and methods

### Animals and ethics statement

Kunming male mice (18-22 g) were obtained from Shanghai Slac Laboratory Animal Corp., Ltd. (certificate no.SCXK (HU) 2012–0002) and bred in our animal facility. The animals were housed in a controlled 12 h dark-light cycle at a temperature of 24 ± 2 °C and a relative humidity of 60 ± 10%, and were provided access to water ad libitum. All procedures were performed in accordance with the protocol outlined in the Guide for the Care and Use of Laboratory Animals, published by the US National Institute of Health (NIH publication no. 85–23, revised 1996) and approved by the Committee on the Ethics of Animal Experiments of Anhui Medical University.

### Drugs and reagents

XJEK is composed of 14 medicinal compositions as presented in Table 1. All herbs were obtained from Hefei Company of Traditional Crude Drugs (Hefei, China), and carefully validated by Dr. He-ping Huang (Anhui University of Traditional Chinese Medicine, Hefei, China). The specimens were stored at the Herbarium of the Department of Pharmacology, Basic College of Medicine, Anhui Medical University (Hefei, China). Fosinopril was purchased from Bristol-Myers Squibb (Shanghai, China), and *L*-NAME was obtained from Sigma-Aldrich; Merck KGaA (Darmstadt, Germany).

### Extraction and isolation

The XJEK decoction was prepared and quality control was performed as described previously [6]. Subsequently, the aqueous polysaccharide fraction was extracted as described previously [20]. Briefly, ~ 3, 380 g extractum were mixed, immersed and boiled in a 10-fold volume of distilled water for 2 h in an automatic boiling machine, which was repeated twice. The solution was then collected, concentrated and freeze-dried to yield a powder at a 1:5 ratio with raw materials. Subsequently, using a rotatory evaporator, XJEK was partitioned with ethyl acetate (EAE;~ 10 l) and n-butanol (BuE;~ 10 l), and this was repeated three times to acquire EAE extracts (9.1 g), BuE extracts (82.8 g) and aqueous layer extracts (AqE; 1114 g), which were freeze-dried for 48 h. All the concentrated AqE extracts were then submitted to trichloroacetic acid precipitation for protein removal to yield polysaccharide extract.

### Cell culture and experiment design

HUVECs were routinely maintained in phenol red-containing Dulbecco’s modified Eagle’s medium (Hyclone; GE Healthcare, Chicago, IL, USA) supplemented with 10% fetal bovine serum (Tianhang Biotechnology Co., Ltd., Zhejiang), in a 37 °C incubator at an atmosphere of 5% CO_2_. The cells were randomly divided into seven groups and incubated for 24 h with the corresponding drugs. The seven groups included a blank control group, an Ang II (10^− 5^ mol/l) group, an Ang II (10^− 5^ mol/l) + XJEK (1.6 mg/ml) group, an Ang II (10^− 5^ mol/l) + AqE (1.2 mg/ml) group, an Ang II (10^− 5^ mol/l) + AqE (0.6 mg/ml) group, an Ang II (10^− 5^ mol/l) + AqE (0.3 mg/ml) group and an Ang II (10^− 5^ mol/l) + AqE (0.15 mg/ml) group. Following treatment, the supernatant was collected to examine NO and endothelial nitric oxide synthase (eNOS) expression levels by Griess method and enzyme linked immunosorbent assay (ELISA), respectively.

### Immunofluorescence

Following the treatment of HUVECs, the supernatant in the 24-well plates was discarded and the cells were washed with 0.01 mol/l PBS (pH 7.4) three times. Subsequently, the cells were fixed with 4% paraformaldehyde (Sigma-Aldrich; Merck KGaA) and blocked. The samples were then incubated with primary eNOS monoclonal antibody (1:100; Abcam, Cambridge, UK) overnight at 4 °C. Following three washes with PBS, FITC-labeled goat anti-mice IgG antibody was added for 60 min, followed by incubation with 5 mg/ml DAPI (Sigma-Aldrich; Merck KGaA) for 5 min in an enamel box at room temperature. Following routine washing, digital pictures were obtained using an Olympus IX71inverted fluorescence microscope (Olympus Cooperation, Tokyo, Japan).

### Induction of hypertension in mice and design of experiment

A total of 50 Kunming mice were randomized into five groups with ten mice in each group and then the mouse hypertension model was induced by the administration of *L*-NAME (2 mg/ml; ~ 160-180 mg/kg/day) in drinking water for a consecutive 8 weeks. A regular chow diet was used to feed all experimental subjects. Group1 was a control group, in which mice were provided tap water alone for 8 weeks. Group2 was a model group, in which mice were given *L*-NAME in drinking water for 8 weeks. Group3 was the *L*-NAME+AqE-treated group, in which mice were given *L*-NAME in drinking water for 8 weeks and intragastric administration of AqE (2.47 g/kg/day) for the last 4 weeks. Group4 was the *L*-NAME+XJEK-treated group, in which mice were given *L*-NAME in drinking water for 8 weeks and intragastric administration of XJEK (7.5 g/kg/day) for the last 4 weeks. Group5 was the *L*-NAME+fosinopril-treated group, in which mice were given *L*-NAME in drinking water for 8 weeks and intragastric administration of fosinopril (2 mg/kg/day) for the last 4 weeks.

### Measurement of systolic blood pressure

Systolic blood pressure (SBP) was measured using the tail-cuff method with an ALC-NIBP blood pressure meter (Shanghai Alcott Biotech Co., Ltd., Shanghai, China). The basal blood pressure of each group was recorded prior to the treatment and once a week during the following 8 weeks of treatment. All measurements were obtained by the same individual at the same time in the day. Prior to measuring the SBP, all mice were warmed for 30 min at 28 °C to obtain a steady pulse level and allow the detection of tail artery pulsations. SBP was determined by an average of ten measurements.

### Haemodynamic parameters and cardiac remodeling index

Following 8 weeks of treatment, all mice were anesthetized with pentobarbital sodium (45 mg/kg IP). The right carotid artery was then cannulated with a catheter (Transonic Scisense Inc., London, ON, Canada), which was connected to an admittance control unit. Subsequently, the catheter was inserted into the left ventricle along the right coronary artery. The signals were recorded on a four-channel acquisition system (BL420S; Chengdu Technology & Market Co., Ltd., Chengdu, China). The admittance catheters were soaked for 30 min in Alconox prior to insertion into the common carotid artery, according to the manufacturer’s protocol. The left ventricular systolic pressure (LVSP), left ventricular end-diastolic pressure (LVEDP) and rate of rise of left ventricular pressure (dp/dtmax) were recorded. Subsequently, blood samples were acquired from the heart and collected into tubes pretreated with heparin. The tubes were then centrifuged at 3500 rpm for 10 min at 4 °C and the supernatant was stored at − 80 °C for future analysis. Cervical exsanguinations were performed on mice, followed by a quick isolation and perfusion of the hearts with ice-cold cardioplegic solution (30 mM KCl in PBS) to arrest the heart in diastole. The heart weight index (HW/BW) was also calculated by dividing the weight of the heart by the total body weight. Lastly, heart samples were separated into several sections for different analyses.

### Isolated vascular ring experiments

Following the opening of thorax, the thoracic aorta underwent instant excision. Subsequently, followed by the removal of loose connective tissues, the transverse ring (~ 4-5 mm in length) was dissected and all vessels were suspended in organ baths as rings bathed in Krebs solution composed of 118 mM NaCl, 4.75 mM KCl, 25 mM NaHCO_3_, 1.2 mM MgSO_4_, 2 mM CaCl_2_, 1.2 mM KH_2_PO_4_, and 11 mM glucose. Krebs solution was maintained at 37 ± 1 °C, and gassed with 95% O_2_ and 5% CO_2_ (pH 7.4). This protocol had been performed previously for mouse aortic strips and 0.5 g of tension was determined as optimal for this tissue. Seven rings underwent equilibration for 60–90 min, and tissues were stretched and washed with warm Krebs solution every 15 min during the process. With intact rings contracted previously with 10^− 5^ M phenylephrine, the concentration-relaxation response curves to acetylcholine (ACh) (10^− 9^-10^− 6^ M) were graphed. Relaxant responses to ACh were expressed as a percentage of the precontraction achieved by phenylephrine.

### Histological and morphological analyses of heart and thoracic aorta

The apex of the mouse heart was immersed in neutral 10% buffered formalin for histological analysis. Paraffin sections (5 μm) were sliced and stained with hematoxylin and eosin (HE) and Van Gieson. Subsequently, the myocyte cross-sectional area (CSA), perivascular collagen area (PVCA) and collagen volume fraction (CVF) were calculated by quantitative analysis of digitalized microscopic images using Image J software (1.4, 3.67). Thoracic aortas obtained from mice were cleaned and maintained in neutral 10% buffered formalin. Subsequently, thoracic aorta sections (5 μm) were embedded in paraffin, cut, dewaxed and then stained with HE. Image-pro plus software (Media Cybernetics, Inc., Rockville, MD, USA) was used to measure the following: Area of total aorta (TAA), area of lumen (LA), CSA, aorta radius (AR), luminal radius (L) and media thickness (M). Meanwhile, the ratio of M/L was calculated as described previously [21].

### Measurement of NO content, eNOS activity and asymmetric dimethylarginine (ADMA) levels in plasma

Due to the short half-life and low concentration of NO in vivo, plasma NO levels were assessed by measuring its stable metabolites, including nitrite and nitrate, which were determined using a NO detection kit (Nanjing Jiancheng Bioengineering Institute, Nanjing, China), according to the manufacturer’s protocol. In addition, eNOS activity and ADMA levels were estimated using ELISA, according to the manufacturer’s protocol.

### Determination of interleukin-1β (IL-1β), tumor necrosis factor-α (TNF-α) and interleukin-10 (IL-10) concentrations in plasma, cardiac tissues and HUVECs

Chronic *L*-NAME induces a systemic inflammation-like condition in mice [22], which could be considered a marker for *L*-NAME-induced hypertension. Therefore, the current study measured the expression levels of IL-1β, TNF-α and IL-10 in plasma, cardiac tissues and supernatants of HUVECs using ELISA, according to the manufacturer’s protocol.

In addition, immunohistochemical staining was performed with antibodies to detect IL-1β, TNF-α and IL-10 expression levels in cardiac tissues, according to the manufacturer’s protocol (Boster Biological Technology, Pleasanton, CA, USA). Briefly, 5-μm sections were incubated with rabbit polyclonal primary antibodies (1:200) overnight at 4 °C.Following three washes, the sections were incubated with secondary antibody for 10 min and then incubated with diaminobenzidine for 5 min, followed by haematoxylin counterstaining. Images werethen obtained using a digital camera system (Olympus BX35; Olympus Cooperation).

### Measurement of total superoxide dismutase (SOD) activities, malondialdehyde (MDA) content in plasma

As has been described previously [23], a thiobarbituric acid reactive substances assay (Jiancheng Institute of Bioengineering Company, Nanjing, China) was used for the detection of MDA by measuring the absorbance at a wavelength of 532 nm, according to the manufacturer’s protocol. The xanthine oxidase method was employed to measure SOD activity by determining the absorbance at 550 nm with a SOD kit (Jiancheng Institute of Bioengineering Company).

### Western blotting analysis

After the cell culture density reached 90%, the cells were treated with the above 2.4 method, and after continuing to culture for 24 h, they were washed with cold PBS, and then lysed with a lysate for 30 min. After centrifugation at 4 °C (12000r·min^− 1^, 10 min), the supernatant was taken, and 20 μl of the supernatant was taken to determine the protein concentration by BCA Protein Assay Kit (Beyotime). Proteins were separated by SDS-PAGE and transferred to PVDF membranes by wet transfer. After closed in 5% nonfat milk solution at 37 °C for 1 h, the membranes were soaked with the following primary antibodies in TBS-T solutions overnight at 4 °C: rabbit anti-eNOS antibody (1:1000, Abcam, USA.) and rabbit anti-GAPDH antibody (1:1000, Affinity, USA.). After incubated with secondary antibodies (goat anti-rabbit IgG, 1:5000; Affinity, USA.), the gray value of each belt was detected using the Super signal enhanced chemiluminescence (ECL; Amersham Biosciences, Little Chalfont, UK) detection system. The relative band intensity was determined using GAPDH as a loading control by ImageJ software.

### Statistical analysis

Results are presented as the mean ± SD. A t-test was used to analyze differences between basal and treatment values in the same set of experiments. One-way analysis of variance with a Tukey-Kramer test was performed for comparison of data among different sets of experiments. In addition, the least-squares method was used for assessment of linear correlation between the selected variables. *P* < 0.05 was considered to indicate a statistically significant difference.

## Results

### Effect of polysaccharide extract from XJEK on NO content, eNOS activity and expression levels in AngII-induced HUVECs

**Fig. 1:**
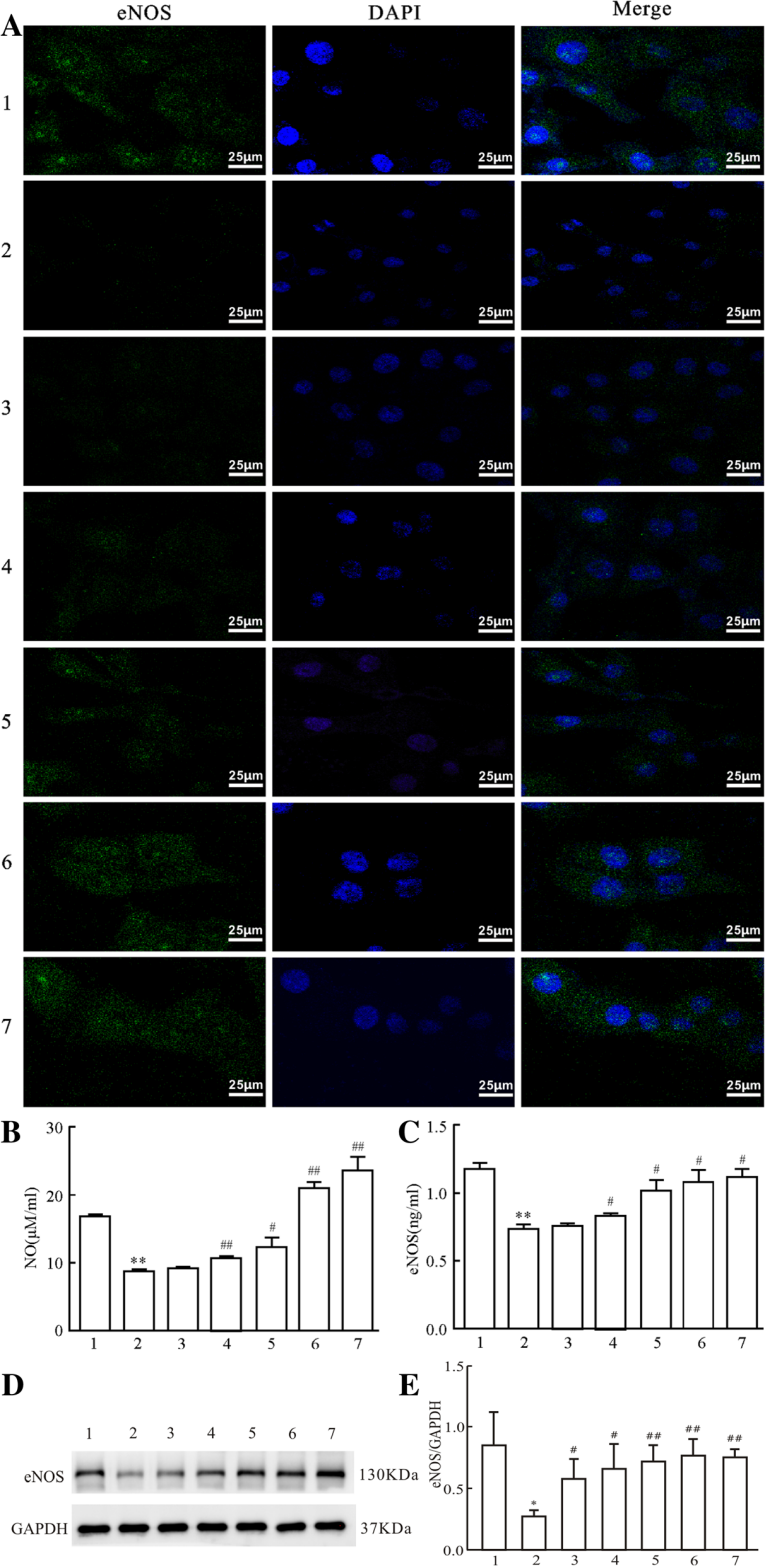
Effects of polysaccharide extract from XJEK on NO content and eNOS expression in Ang II-induced HUVECs. (**a**): eNOS expression in HUVECs by immunofluorescence analysis (magnification, × 400). (**b**): NO content in supernatants of HUVECs; (**c**): eNOS activity in supernatants of HUVECs (ELISA assay); (**d**): eNOS expression in HUVECs by Western blot analysis and (**e**) statistical analysis of eNOS protein expression. 1, blank control group; 2, Ang II (10^− 5^ mol/l) group; 3, Ang II (10^− 5^ mol/l) + AqE (0.15 mg/ml) group; 4, Ang II (10^− 5^ mol/l) + AqE (0.3 mg/ml) group; 5, Ang II (10^− 5^ mol/l) + AqE (0.6 mg/ml) group; 6, Ang II (10^− 5^ mol/l) + AqE (1.2 mg/ml) group; 7, Ang II (10^− 5^ mol/l) + XJEK (1.6 mg/ml) group. Data are presented as the mean ± SD (*n* = 3). ^**^P < 0.01 vs. control group; ^*#*^*P* < 0.05, ^##^*P* < 0.01*vs*. Ang II group

An immunofluorescent microscope was used to detect the eNOS distribution and immunofluorescence intensity in HUVECs. The eNOS protein was predominantly expressed in the cytoplasm of HUVECs and the positive signals presented as a green spotlight with diffused distribution. A statistically significant difference (*P* < 0.05) was identified between the fluorescence intensity values of eNOS protein in the blank control group and the AngII-treated group, which were 169.41 ± 10.70 and 62.01 ± 4.43, respectively. Treatment with AqE could markedly increase eNOS expression in a dose-dependent manner (Fig. 1 a).

Following treatment of HUVECs, the supernatants were harvested, and NO content and eNOS activity were detected. Compared with the blank control group, the NO content and eNOS activity in the AngII-treated group significantly decreased, which could be reversed by treatment with AqE in a dose-dependent manner (Fig. 1c).

We probed into the effect of XJEK on the expression of eNOS in HUVECs by Western bolt. Compared to blank control group, the eNOS expression was apparently lower in Ang II group (*P*<0.05). But Treatment with AqE increased the eNOS protein expression significantly (*P*<0.05). Treatment with XJEK also markedly upregulated the eNOS expression similarly to that by AqE treatment (Fig. 1 d, e).

### Effects of polysaccharide extract from XJEK on TNF-α, IL-1β and IL-10 of HUVECs induced by Ang II

**Fig. 2:**
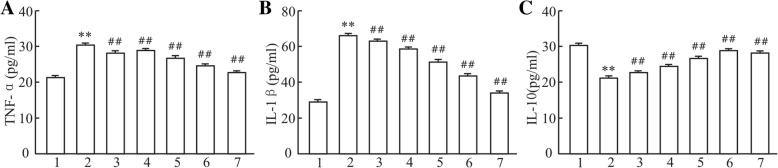
Effects of polysaccharide extract from XJEK on TNF-α, IL-1β and IL-10 of HUVECs induced by Ang II. (**a**) TNF-α level in supernatants of HUVECs; (**b**) IL-1β level in supernatants of HUVECs; (**c**) IL-10 level in supernatants of HUVECs. 1, blank control group; 2, Ang II (10^− 5^ mol/L) group; 3, Ang II (10^− 5^ mol/L) + AqE (0.15 mg/ml) group; 4, Ang II (10^− 5^ mol/L) + AqE (0.3 mg/ml) group; 5, Ang II (10^− 5^ mol/L) + AqE (0.6 mg/ml) group; 6, Ang II (10^− 5^ mol/L) + AqE (1.2 mg/ml) group; 7, Ang II (10^− 5^ mol/L) + XJEK (1.6 mg/ml) group. Data are expressed as mean ± SD, *n* = 6. ^**^P < 0.01 vs control group; ^##^*P* < 0.01 vs Ang II group

We examined the protein expression of pro-inflammatory cytokines (TNF-α and IL-1β) as well as anti-inflammatory cytokines (IL-10) by ELISA kits. As exhibited in Fig. 2, TNF-α and IL-1β were markedly elevated in AngII group compared to those in blank control group (*P* < 0.01). In contrast, after administration of AqE, the protein expression of IL-1β and TNF-α was significantly decreased in supernatants of HUVECs compared to AngII group, and the similar results were observed with XJEK treatment (*P* < 0.01). In addition, we also assayed the protein expression of IL-10 and hereby found that IL-10 level was persistently down-regulated in AngII group. The protein expression of IL-10 in HUVECs with AqE administration was significantly increased compared to that of AngII group (*P* < 0.01).

### Effects of polysaccharide extract from XJEK on SBP in L-NAME-induced hypertensive mice

**Fig. 3:**
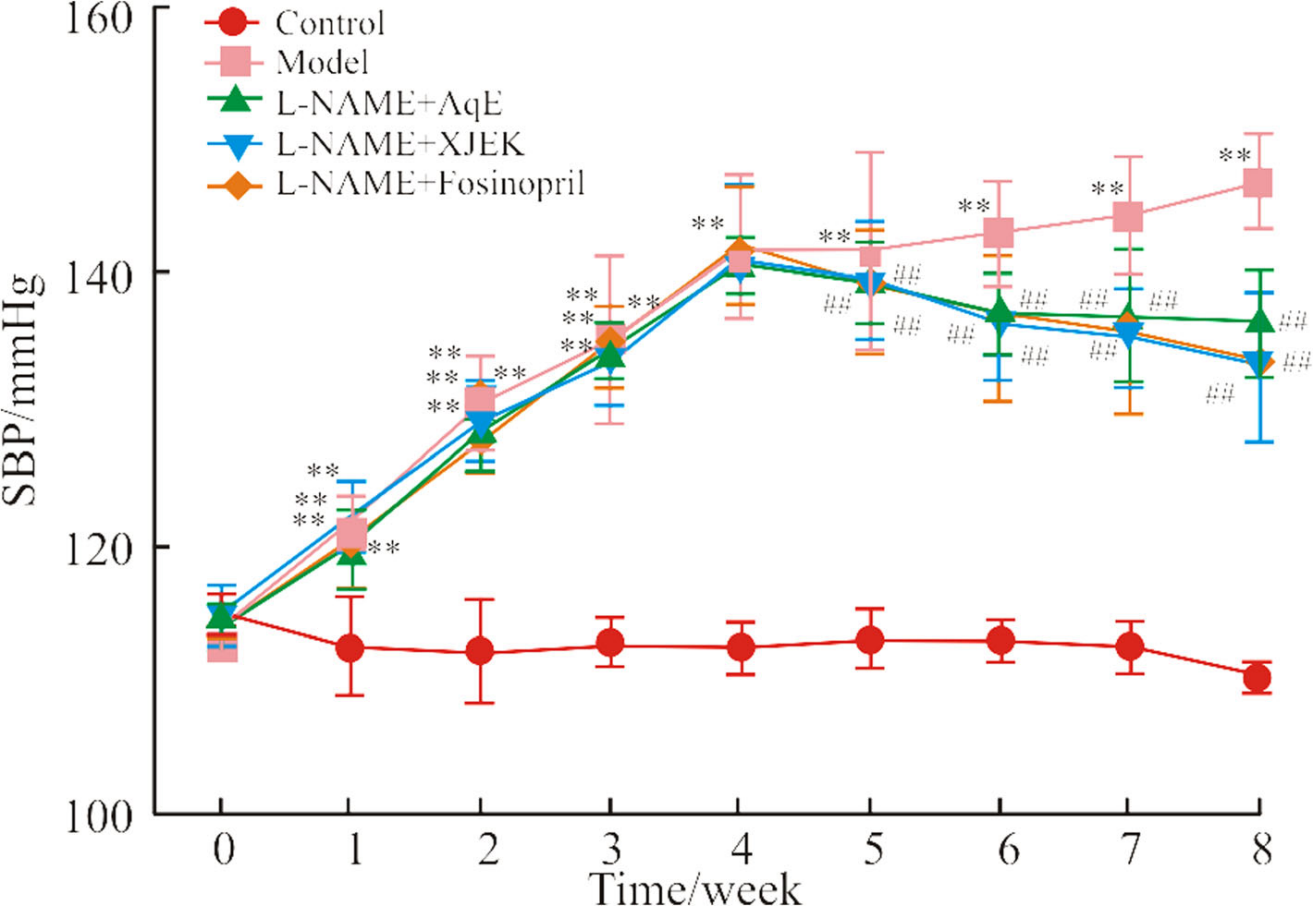
Development of SBP in five groups in an 8-week period. SBP was measured in each group at nine time points using tail-cuff apparatus. The hypertensive response to *L*-NAME administration was time-dependent, reaching a maximum in week 8. AqE was efficacious in inhibiting the development of high blood pressure at week 5 of treatment with *L*-NAME, which was significantly different compared with the model group. Data are presented as the mean ± SD (*n* = 10). ^**^P < 0.01 vs. control group; ^##^*P* < 0.01vs. model group

Time-associated changes in the SBP of the five experimental mice groups are demonstrated in Fig. 3. At baseline, no significant differences were identified between the SBP so fall experimental groups. However, a daily administration of *L*-NAME for 8 weeks resulted in a significant increase in SBP (146.83 ± 3.46 mmHg) compared with that of the control group (110.45 ± 1.09 mmHg) (*P* < 0.01). In addition, treatment with polysaccharide for the last 4 weeks significantly reduced the SBP (133.48 ± 4.45 mmHg).

### Effects of polysaccharide extract from XJEK on haemodynamic parameters in L-NAME-induced hypertensive mice

In vivo left ventricular function in all experimental mice groups was assessed for 8 weeks. As presented in Table 2, the systolic cardiac parameters, LVSP, LVEDP, +dp/dt_max_, and diastolic cardiac parameter -dp/dt_max_ all increased in the *L*-NAME-treated group compared with the control group, however, these increases could be prevented with the administration of polysaccharide or fosinopril.

**Table 2:** Effects of AqE on cardiac function in *L*-NAME induced hypertensive mice (mean ± SD, *n* = 10)

Group	LVSP(mmHg)	LVEDP(mmHg)	+dp/dt_max_(mmHg/s)	-dp/dt_max_(mmHg/s)
Control	89.25 ± 13.02	−0.31 ± 1.79	4616.92 ± 713.09	− 3855.15 ± 659.27
Model	114.5 ± 15.16^**^	−1.15 ± 6.09	5487.19 ± 674.56^*^	− 4767.96 ± 934.34^*^
*L*-NAME+AqE	116.76 ± 9.96	−0.09 ± 8.25	4777.74 ± 797.27	− 3502.52 ± 976.41^##^
*L*-NAME+XJEK	111.41 ± 15.36	−1.18 ± 5.13	4873.61 ± 1113.51	− 3849.29 ± 1039.70
*L*-NAME+Fosinopril	100.56 ± 21.72^#^	0.37 ± 5.32	4618.46 ± 339.42^##^	− 3457.26 ± 736.12^##^

*LVSP* left ventricular systolic pressure, *LVEDP* left ventricular end-diastolic pressure, +dp/dt_max_ maximal rate of left ventricular systolic pressure, −dp/dt_max_ maximal rate of left ventricular diastolic pressure. ^*^P < 0.05, ^**^P < 0.01 vs control; ^#^*P* < 0.05, ^##^*P* < 0.01 vs model

### Effects of polysaccharide extract from XJEK on cardiac remodeling in L-NAME-induced hypertensive mice

**Fig. 4:**
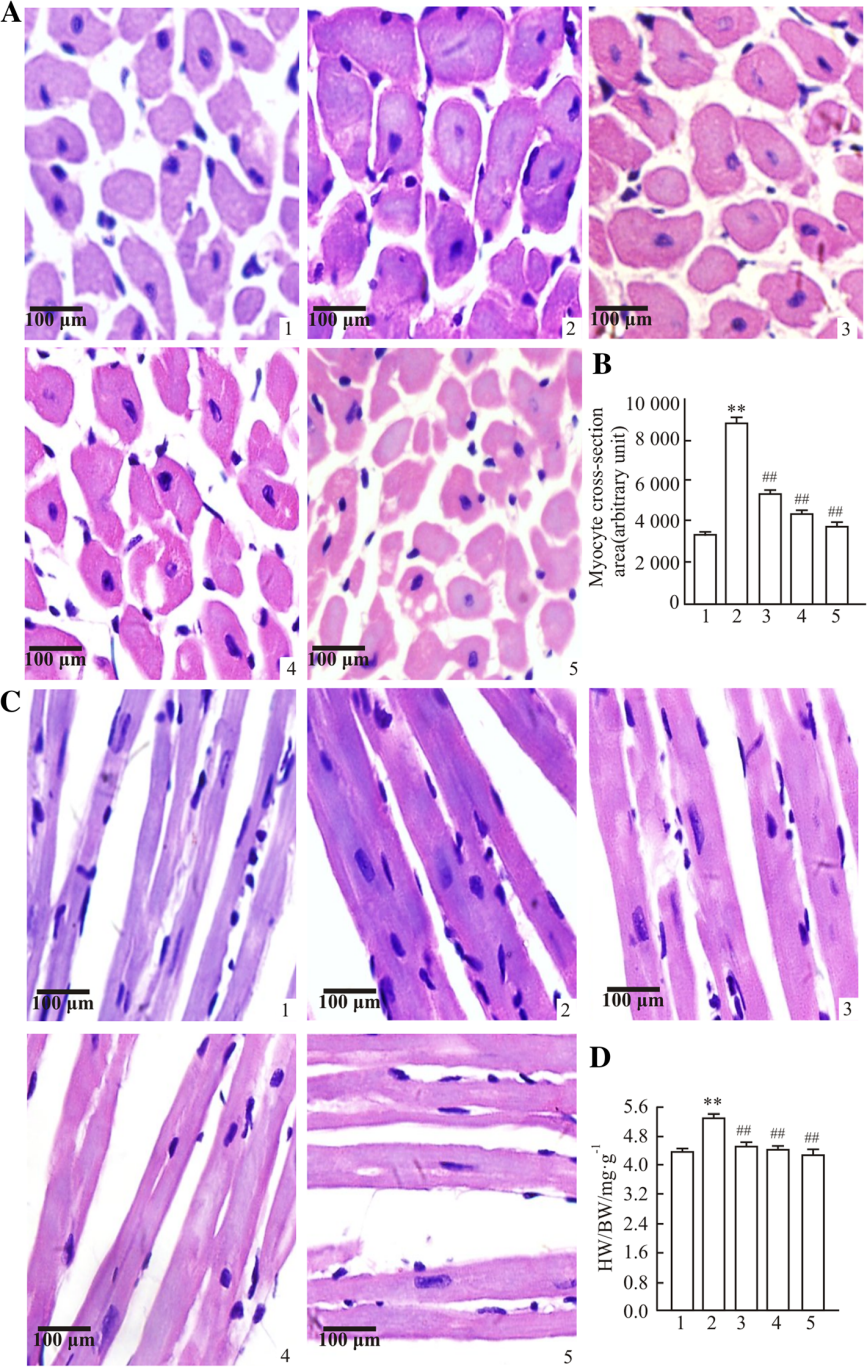
Effects of polysaccharide extract from XJEK on HW/BW and cardiomyocyte CSA in *L*-NAME-induced hypertensive mice. (**a**) Representative image of a cardiomyocyte cross-section following HE staining (magnification, × 400). (**b**) Statistic alanalysis of cardiomyocyte CSA. (**c**) Representative image of a cardiomyocyte long axis following HE staining (magnification, × 400). (**d**) Statistical analysis of HW/BW. 1, Control group; 2,model group; 3,*L*-NAME+AqE group; 4,*L*-NAME+XJEK group; 5,*L*-NAME+fosinopril group. The HW/BW ratios and cardiomyocyte CSAs evidently increased in the model group compared with the control group. Subsequently, following 4 weeks of treatment with polysaccharide, the pathological changes were reversed, which could similarly be achieved with the positive control fosinopril. Data are presented as the mean ± SD (*n* = 10). ^*^P < 0.05, ^**^P < 0.01 vs. control group; ^##^*P* < 0.01 vs. model group

**Fig. 5:**
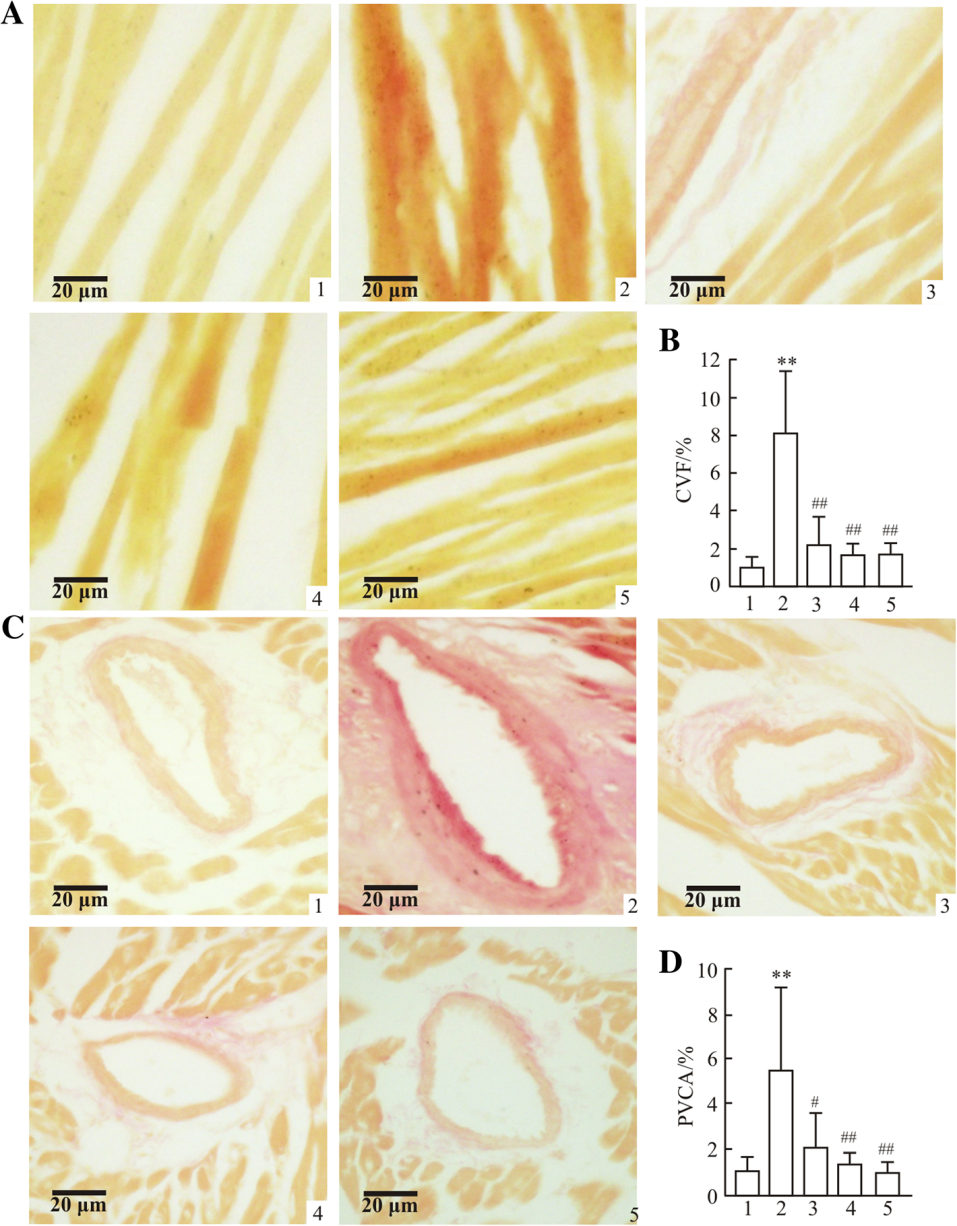
Effects of polysaccharide extract from XJEK on CVF and PVCA in *L*-NAME-induced hypertensive mice. Representative image of myocardial fibrosis following VG staining (magnification, × 400). (**b**) Statistic alanalysis of myocardial fibrosis. (**c**) Representative image of perivascular fibrosis following VG staining (magnification, × 400). (**d**) Statistical analysis of perivascular fibrosis. 1,control group; 2,model group; 3,*L*-NAME+AqE group; 4,*L*-NAME+XJEK group; 5,*L*-NAME+fosinopril group. Levels of myocardial and perivascular fibrosis increased significantly in the model group compared with control group. However, these pathological changes could be reversed following AqE for the last 4 weeks of treatment and similar effects could be achieved with treatment with the positive control fosinopril. Data are presented as the mean ± SD (*n* = 10). ^**^P < 0.01 vs. control group; ^#^*P* < 0.05, ^##^*P* < 0.01 vs. model group

Cardiac hypertrophy is associated with an elevated HW/BW ratio. Compared with the control group, the HW/BW ratios markedly increased in the treatment groups (Fig. 4d). Histological investigation of hearts obtained from mice in the hypertensive model group revealed an evident increase in cardiomyocyte CSA, CVF and PVCA compared with the control group (*P* < 0.01; Fig. 4a-c and Fig. 5a-d). However, treatments with polysaccharide or fosinopril for 4 weeks were revealed to reverse these pathological changes.

### Effects of polysaccharide extract from XJEK on aortic remodeling in L-NAME-induced hypertensive mice

**Fig. 6:**
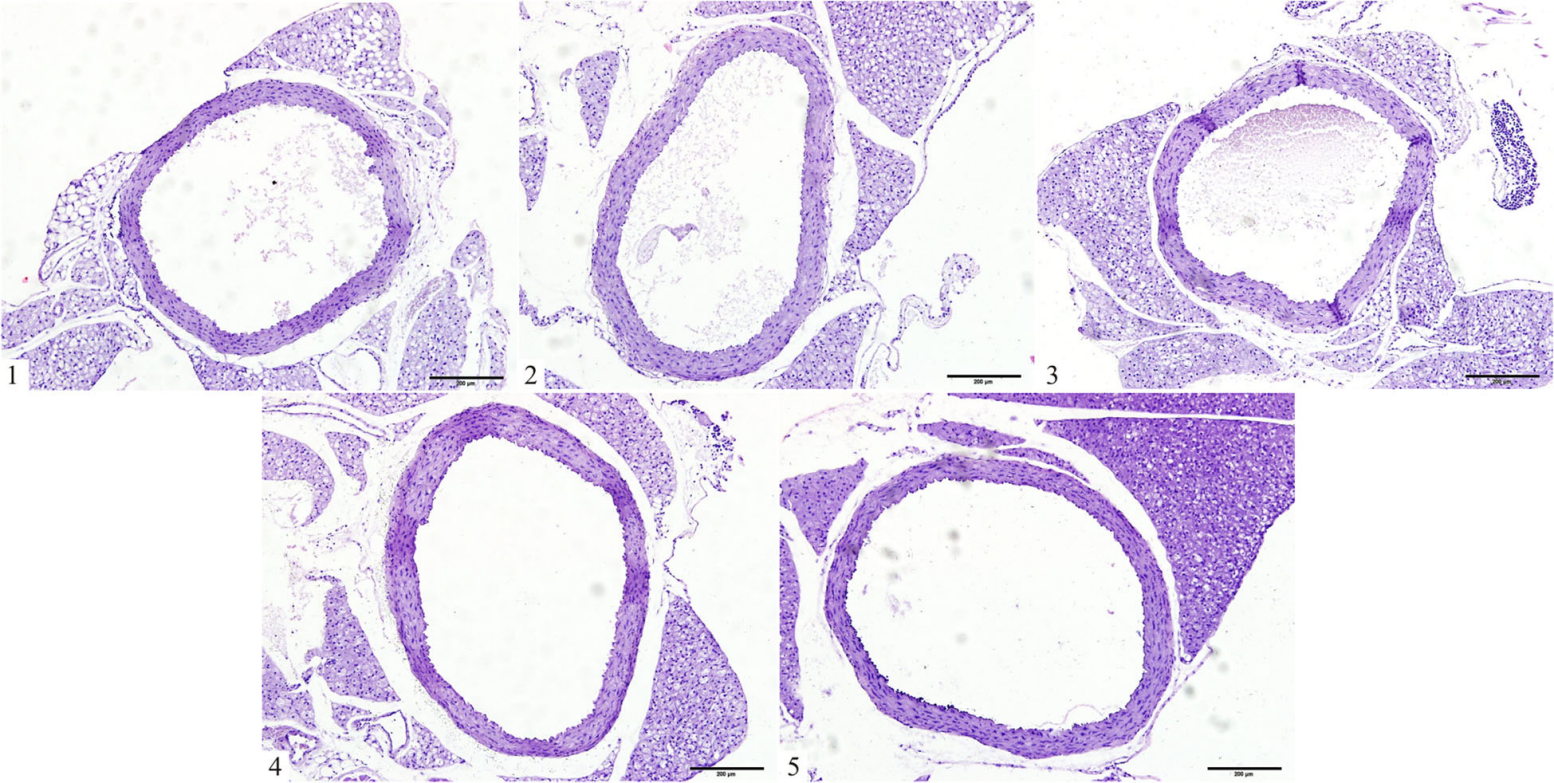
Representative images of aortic remodeling in different groups following HE staining (magnification, × 40). 1,control group; 2,model group; 3,*L*-NAME+AqE group; 4,*L*-NAME+XJEK group; 5,*L*-NAME+fosinopril group. The model group demonstrated notable vascular remodeling compared with the control group. However, these pathological changes could be markedly reversed following treatment with AqE for the last 4 weeks of treatment, and similar effects could be achieved when XJEK and the positive control fosinopril were administered

Following 8 weeks of treatment, observations were made regarding vascular remodeling of the upper thoracic aorta in mice exposed to *L*-NAME. TAA, LA, CSA, AR, M and M/L ratio values were revealed to be significantly higher in *L*-NAME-treated mice compared with the control group. However, this effect of *L*-NAME was inhibited by treatments with AqE or fosionporil for the last 4 weeks (Table 3; Fig. 6).

**Table 3:** Effects of AqE on aorta remodeling in *L*-NAME induced hypertensive mice (mean ± SD, *n* = 10)

Group	TAA(×10^3^μm^2^)	LA(×10^3^μm^2^)	CSA(×10^3^μm^2^)	CSA/TAA(%)	AR(μm)	Lumen(μm)	Media(μm)	M/L(%)
Control	424.5 ± 81.2	271.7 ± 66.6	152.8 ± 17.9	36.0 ± 22.0	366.4 ± 33.8	292.5 ± 33.9	73.9 ± 4.6	25.5 ± 3.0
Model	686.8 ± 162.3^**^	426.4 ± 97.1^**^	260.3 ± 73.1^**^	27.9 ± 45.0	464.8 ± 56.7^**^	366.3 ± 44.3^**^	98.5 ± 17.6^**^	27.0 ± 3.8
*L*-NAME+AqE	496.6 ± 81.5^#^	319.0 ± 59.6^#^	177.6 ± 22.0^#^	35.8 ± 27.0	396.5 ± 33.5^#^	317.5 ± 30.7	78.9 ± 2.9^#^	25.0 ± 1.7
*L*-NAME+XJEK	499.9 ± 65.2^#^	327.1 ± 59.5	172.9 ± 22.2^#^	34.6 ± 34.0	398.3 ± 26.7^#^	321.6 ± 29.4	76.6 ± 9.6^#^	24.1 ± 4.2
*L*-NAME+Fosinopril	445.1 ± 220.2^#^	292.5 ± 148.9^#^	153.2 ± 726.7^#^	34.4 ± 33.0	336.3 ± 159.0^#^	272.1 ± 129.2^#^	64.7 ± 30.0^#^	22.1 ± 6.5

*TAA* total aorta area, *LA* lumen area, *CSA* cross-sectional area, *AR* aorta radius. The vascular remodeling of the upper thoracic aorta exposed to *L*-NAME was observed at the end of 8th week, which could be prevented by treatment with AqE for the last 4 weeks, as well as with two positive drugs, XJEK and fosinopril. Data are expressed as mean ± SD, *n* = 10. ^**^P < 0.01 vs control; ^#^*P* < 0.05 vs model

### Effects of AqE on endothelial dysfunction in L-NAME-induced hypertensive mice

**Fig. 7:**
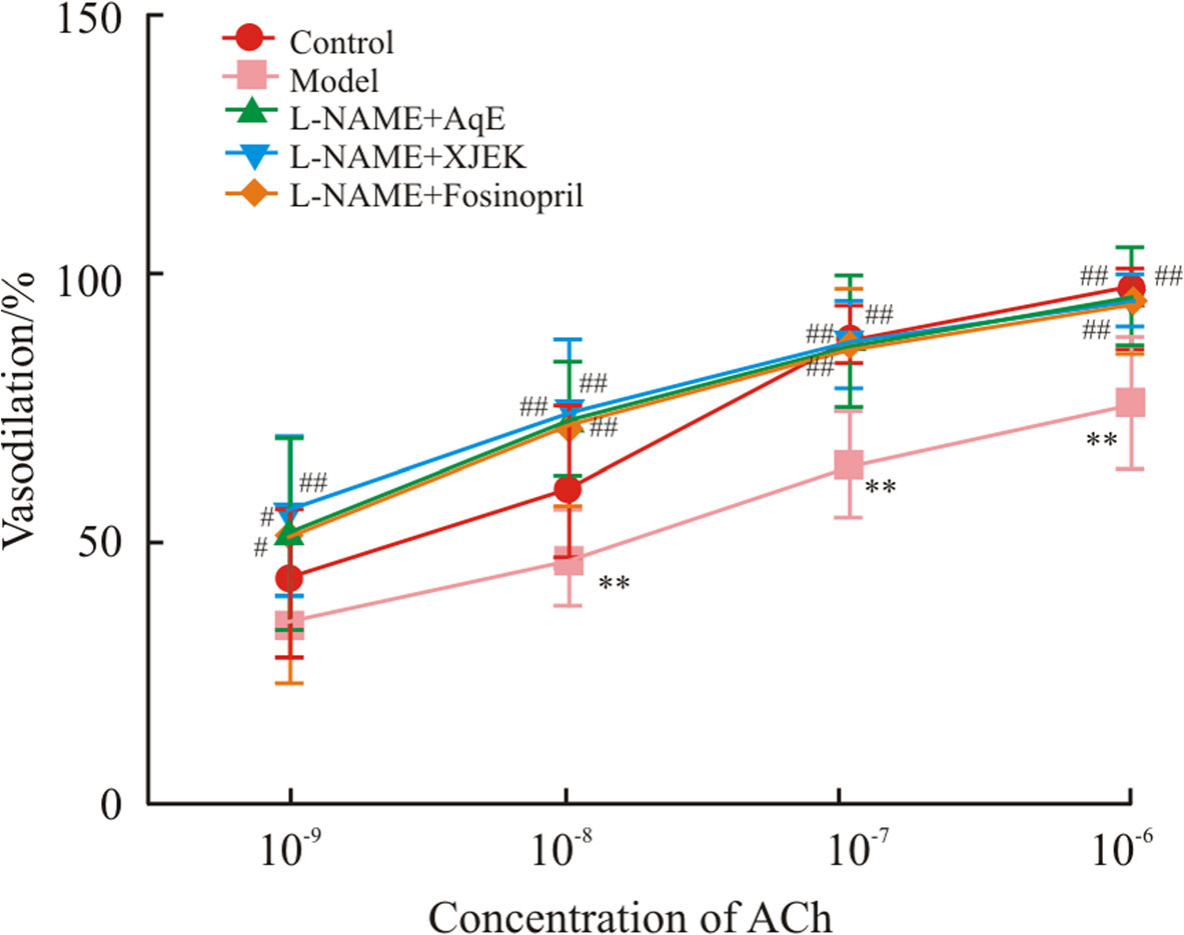
Effects of polysaccharide extract from XJEK on ACh-induced endothelium dependent relaxation. In a phenylephrine pre-contracted isolated mouse aortic ring preparation, administration of ACh induced notable endothelium-dependent relaxation in a dose-dependent manner, which was markedly lower in aorta isolated from *L*-NAME-induced hypertensive mice. A significant restoration of ACh-induced endothelium-dependent relaxation was observed in aorta isolated from polysaccharide-treated hypertensive mice. Data are presented as the mean ± SD (*n* = 10). ^**^P < 0.01 vs. control group; ^#^*P* < 0.05, ^##^*P* < 0.01 vs. model group

Compared with the control aortic rings, phenylephrine-stimulated aortic rings from *L*-NAME-treated mice demonstrated markedly decreased endothelium-dependent vasodilator responses to ACh (Fig. 7). However, the aortic rings from *L*-NAME- and polysaccharide-treated mice revealed a marked increase in ACh-induced vasodilatation compared with the aortic rings from mice treated with *L*-NAME alone.

### Effects of polysaccharide extract from XJEK on plasma NO content, eNOS activity and ADMA level in L-NAME-induced hypertensive mice

NO content and eNOS activity in plasma from *L*-NAME treated mice were significantly lower compared with the control group. However, mice treated with AqE or fosinopril, in addition to *L*-NAME, demonstrated increased expression of NO and increased eNOS activity compared with mice treated with *L*-NAME alone. ADMA is a small molecule amino acid substance that affects vascular activity and vascular endothelial cell function via inhibition of NO synthesis. Compared with the control group, plasma levels of ADMA were significantly higher in the *L*-NAME-treated group. However, treatment with AqE for the last 4 weeks of treatment markedly decreased plasma ADMA levels compared with the *L*-NAME-treated group (Table 4.).

**Table 4:** Effects of AqE on endothelial dysfunction in *L*-NAME induced hypertensive mice

Group	NO(μmol·L^−1^)	eNOS(U·L^−1^)	ADMA(nmol·L^−1^)
Control	22.86 ± 30.35	27.44 ± 3.47	333.87 ± 57.42
Model	1.66 ± 3.11^**^	17.21 ± 3.09^**^	486.31 ± 41.18^**^
*L*-NAME+AqE	7.98 ± 9.64^#^	20.68 ± 1.81^#^	459.26 ± 42.08^#^
*L*-NAME+XJEK	19.59 ± 12.83^##^	22.86 ± 2.43^#^	425.02 ± 49.95^#^
*L*-NAME+Fosinopril	6.73 ± 5.81^#^	22.39 ± 2.80^##^	442.14 ± 46.02^#^

Data are expressed as mean ± SD, *n* = 10. ^*^P < 0.05, ^**^P < 0.01 vs control group; ^#^*P* < 0.05, ^##^*P* < 0.01 vs model group

### Effects of polysaccharide extract from XJEK on inflammation-associated cytokine levels inserum and cardiac tissues of L-NAME-induced hypertensive mice

**Fig. 8:**
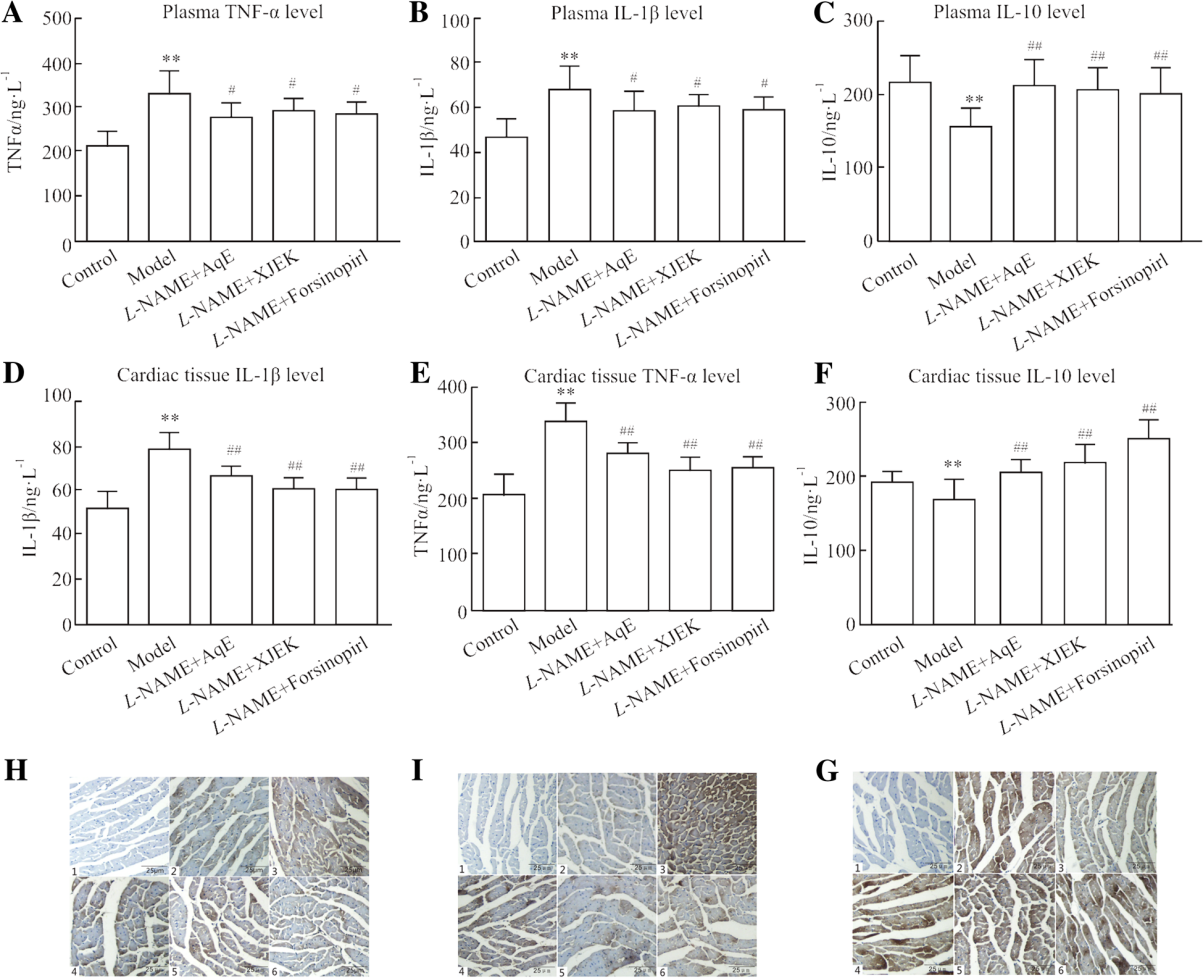
Effects of polysaccharide extract from XJEK on TNF-α, IL-1β and IL-10 in *L*-NAME-induced hypertensive mice. (**a**) TNF-α expression level in plasma. (**b**) IL-1β expression level in plasma. (**c**) IL-10 expression level in plasma. (**d**) IL-1β expression level in cardiac tissues. (**e**) TNF-α expression level in cardiac tissues. (**f**) IL-10 expression level in cardiac tissues. (**g**) Representative image of IL-1β immunocytochemistry. (**h**) Representative image of TNF-α immunocytochemistry.(**i**) Representative image of IL-10 immunocytochemistry.1,negative group; 2,control group; 3, model group; 4,*L*-NAME+AqE group; 5,*L*-NAME+XJEK group; 6,*L*-NAME+fosinopril group. Data are presented as the mean ± SD (*n* = 10).^**^P < 0.01 vs. control group; ^#^*P* < 0.05, ^##^*P* < 0.01 *vs,* model group

The levels of TNF-α and IL-1β in plasma and cardiac tissues were significantly higher in the *L*-NAME-treated group compared with the control group. However, these increased levels could be prevented by treatment with AqE (Fig. 8a,b,d,e). In *L*-NAME induced hypertensive mice, the levels of IL-10 in plasma and cardiac tissues were reduced significantly compared with the control group, however, the levels of IL-10 were markedly higher in mice treated with AqE compared with those treated with *L*-NAME alone (Fig. 8c and f). Immunohistochemistry staining for IL-1β and TNF-α in cardiac section (Fig. 8g and h) indicated evidently higher IL-1β and TNF-α protein expression levels in cardiac sections from *L*-NAME-treated mice compared with the control group, however, treatment with AqE inhibited this *L*-NAME-induced increase of IL-1β and TNF-α expression levels in cardiac tissues. Additionally, in *L*-NAME-induced hypertensive mice, the expression level of IL-10 protein was lower in cardiac sections compared with the control group, however, this decrease in expression level was inhibited by AqE (Fig. 8i).

### Effects of polysaccharide extract from XJEK on plasma SOD activity and MDA content in L-NAME-induced hypertensive mice

Plasma SOD activity markedly decreased in the *L*-NAME-treated group compared with the control group. By contrast, plasma MDA evidently increased in the *L*-NAME-treated group compared with the control group. However, treatment with polysaccharide for the last 4 weeks of treatment markedly downregulated the content of MDA and upregulated plasma SOD activity (Table 5).

**Table 5:** Effects of AqE on SOD and MDA content in *L*-NAME induced hypertensive mice

Group	MDA(μmol·L^−1^)	SOD(U·L^− 1^)
Control	0.50 ± 0.09	242.00 ± 18.89
Model	1.25 ± 0.36^*^	159.38 ± 18.86^**^
*L*-NAME+AqE	0.77 ± 0.13^#^	180.77 ± 13.66^#^
*L*-NAME+XJEK	0.74 ± 0.20^#^	187.22 ± 22.55^#^
*L*-NAME+Fosinopril	0.90 ± 0.05^#^	199.25 ± 24.06^##^

Data are expressed as mean ± SD, *n* = 10. ^*^P < 0.05, ^**^P < 0.01*vs* control group; ^#^*P* < 0.05, ^##^*P* < 0.01 vs model group

## Discussion

**Fig. 9:**
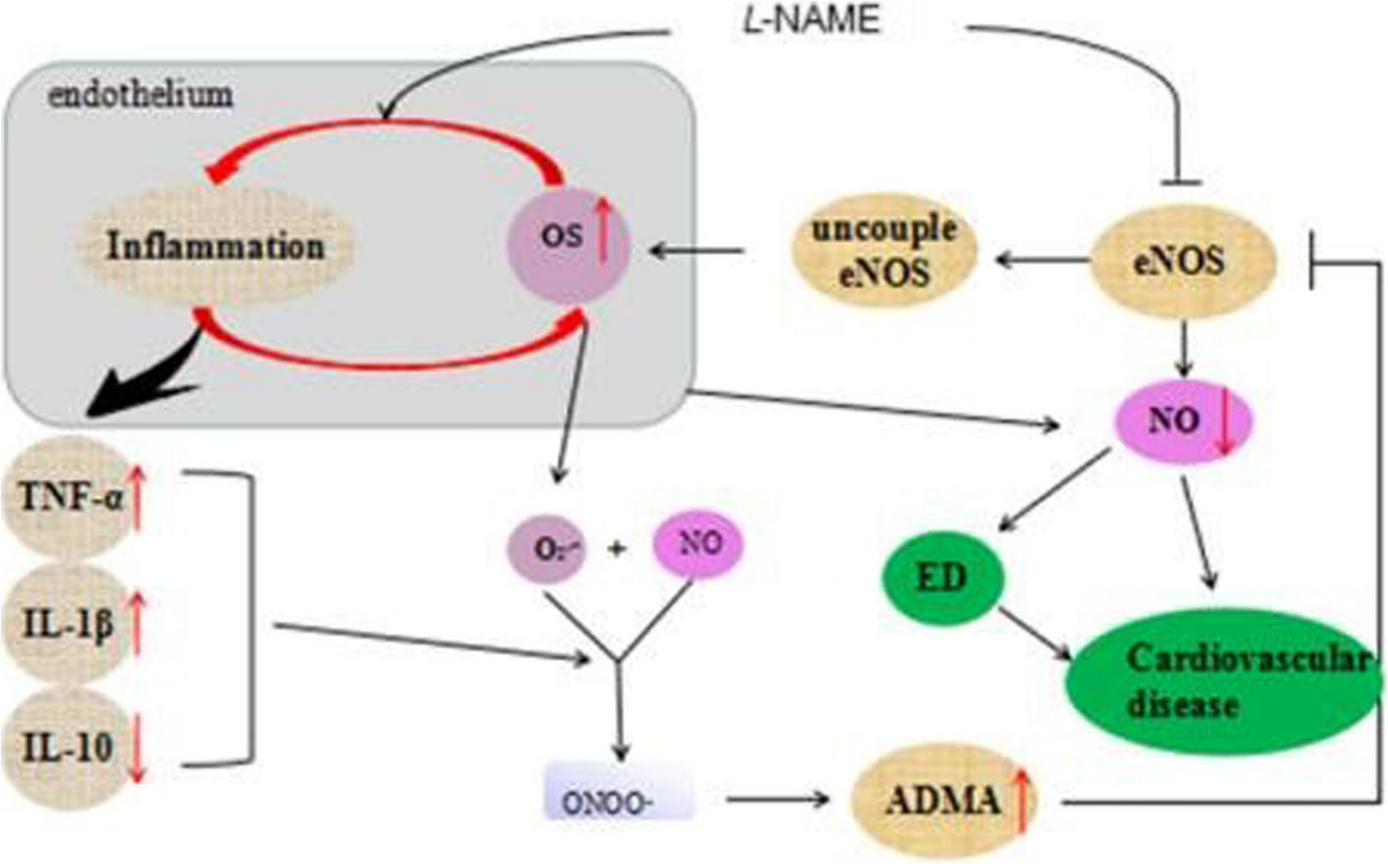
Summary of manuscript

Long-term administration of *L*-NAME in drinking water is a commonly used method for the establishment of chronic hypertension models, in which endothelial dysfunction, increased ventricular hypertrophy, elevated oxidative stress, decreased eNOS expression levels, increased plasma ADMA expression levels and an increased inflammatory status are typical characteristics, as summarized in Fig. 9. The findings in the present study validated the effects of *L*-NAME administration on BP, ED, cardiac hypertrophy, vascular remodeling and inflammatory status, as previously reported. Furthermore, it was demonstrated that treatment with AqE reduces these pathophysiological changes, as does treatment with the positive control drug fosinopril.

In recent years there has been a novel but increasingly important trend of studies investigating the modernization of TCM to achieve a lower dose requirement with the same efficacy, which has predominantly been attempted by the elimination of impurities and the guarantee of active ingredients in compound preparations of TCM. Notably, both clinical and basic studies have demonstrated the efficacy of polysaccharides extracted from *Panax ginseng* C.A.Mey. [24], *Polygonatum odoratum* (Mill.)Druce [25], *Astragalusmongholicus* Bunge [26] and *Ophiopogon japonicus* (Thunb.) Ker Gawl [27] in the prevention of cardiovascular diseases and associated disorders. Specifically, a randomized controlled trial demonstrated that *Panax ginseng* C.A.Mey. polysaccharide may be an effective natural alternative for enhancing the immune system [28]. Zhang et al [20] reported that *Panax ginseng* C.A.Mey polysaccharide can improve pressure overload-induced cardiac remodeling by protecting mitochondria function and reducing energy metabolism dysfunction. The anti-hypertensive function of *Astragalusmongholicus* Bunge polysaccharide has been identified to be associated with an enhancement of endothelial function and anti-inflammation [29–31]. Similarly, in clinical reports byJiang [32] and Luan [33], *Astragalusmongholicus* Bunge polysaccharide was associated with a reduction of the haemodynamic index and cardiac fibrosis in spontaneous hypertension rats (SHR) and in isoproterenol-induced cardiac hypertrophy. Shenmai injection, also known as a traditional Chinese herbal medicine extraction, has gained popularity in China in therapies targeting chronic heart failure, hypertension, and angina pectoris [34, 35]. Furthermore, *Ophiopogon japonicus* (Thunb.) Ker Gawl polysaccharide has been demonstrated to exhibit anti-myocardial ischemic activities in studies by of the Ph. Xu D. S (Shanghai University of Traditional Chinese Medicine) group [36, 37]. Recent studies by Chen [38] and Gu [39] indicateda strong anti-oxidative effect of polysaccharide from *Polygonatum odoratum* (Mill.) Druce for the treatment of metabolic disorders. *Polygonatum odoratum* (Mill.) Drucehas been used for nutritional and medicinal purposes for over 2000 years in China, however, a limited number of studies have investigated its role and pharmacological mechanisms. In accordance with the aforementioned studies, the present study demonstrated an expected effect of AqE and this effect was observed at a lower dose compared with that of XJEK (2.47 g/kg vs. 7.5 g/kg). The pathological characteristics of the animal models used in the current study may explain the similar effect of AqE.

eNOS inhibition induces thickening of the aorta in hypertensive mice, which impacts the elasticity of the aorta vessel wall [40, 41]. A recent study reported an early 50% increase in fibrosis surrounding the aortas of *L*-NAME-treated mice compared with those of untreated mice [42]. ADMA regulates eNOS activity, which results in decreased levels of NO synthesis and increased generation of superoxide [43]. ADMA activity is also susceptible to reactive oxygen and nitrogen species [44]. Therefore, increased systemic ADMA levels may contribute to the pathogenesis and progression of cardiovascular diseases that are associated with endothelial dysfunction. These effects were detected in the current study by the observation of impaired NO mediated responses to ACh in aorta, in addition to decreased NO content and eNOS activity, and increased ADMA consent in plasma and cardiac remodeling. Treatment with AqE for 4 weeks improved cardiovascular remodeling, which was indicated by the restoration of cardiac haemodynamics and an improved HW/BW index, cardiomyocyte CSA, aorta wall thickness, TAA and M. Furthermore, 4-week experimental therapy with AqE alleviated *L*-NAME-induced ED, as indicated by the enhancement of NO-dependent artery relaxation and the restoration of NO content, ADMA content and eNOS activity in plasma. In addition, the present in vitro study demonstrated marked protective effects of AqE on AngII-induced injury of HUVECs, which was indicated by increased NO content and eNOS activity in a dose-dependent manner.

TNF-α interacts with its receptors and in turn activates multiple signals, including the activation of NADPH oxidase. Subsequently, NADPH oxidase induces superoxide production, which reacts with NO and produces the strong oxidant peroxynitrite. In addition, TNF-α inhibits the eNOS promoter and induces the destabilization of eNOS mRNA, which ultimately downregulates the levels of eNOS protein and the ability of the endothelium to produce NO [45, 46]. The aorta ring experiment in the current study demonstrated that TNF-α reduces endothelium-dependent vasodilatation. TNF-α knockout mice may inhibit BP elevation and left ventricular hypertrophy [47]. Furthermore, Mauno et al [48] reported that baseline levels of IL-1β are markedly increased in hypertensive individuals, which suggests a critical role of IL-1β in hypertension. Cytokinesare understood to exhibit anti-inflammatory and anti-hypertensive effects. Didion et al demonstrated an impairment of endothelial dependent vasodilatation in IL-10 knockout mice treated with Ang II. In addition, increased function of Tregs decreases the expression of Ang II and improves aldosterone-induced hypertension, cardiac fibrosis, coronary inflammation, electric remodeling and endothelial-dependent vasodilation, which are likely partly mediated by the release of IL-10 from Tregs [49–52].

Furthermore, NO regulatesthe endogenous production of IL-10, which maintains homeostasis in healthy individuals. It has been suggested that NO may sustain the endogenous levels of IL-10 by promoting the proliferation and survival of Treg cells [53]. Therefore, a balance between pro-inflammatory and anti-inflammatory cytokines is important for cardiac remodeling, hypertension-associated ED and myocardial infarction injury, and the restoration of this balance may prevent cardiovascular damage [54]. It has been reported that polysaccharides perform potent immune-modulatory activities, as demonstrated by the induction of pro-inflammatory and anti-inflammatory cytokines [55]. In the present study an immunological imbalance was revealed in an *L*-NAME-induced hypertensive mouse model, indicated by increased expression levels of TNF-α and IL-1β, and decreased expression of IL-10. Furthermore, the balance between pro-inflammatory and anti-inflammatory status could be restored by treatment with polysaccharide from XJEK (Fig. 9).

## Conclusion

In summary, the current study demonstrated that the administration of polysaccharide from XJEK reduces hypertensive damage in an *L*-NAME-induced hypertensive mouse model by improving endothelial dysfunction and restoring immunologic balance. This suggests a possible protective therapeutic effect of polysaccharide from XJEK by the attenuation of cardiovascular remodeling and ED. However, further studies are required to uncover the mechanisms associated with immunological regulation by XJEK and validate its potential use in therapy.

## Data Availability

The datasets used and/or analysed during the current study are available from the corresponding author (Shan Gao) on reasonable request.

## Abbreviations

±dp/dtmax: Maximal rate of left ventricular systolic and diastolic pressure
Ang II: Angiotensin II
AqE: Aqueous layer extracts
AR: Aorta radius
ASBP: Artery systolic blood pressure
CSA: Cross-sectional area
CVF: Collagen volume fraction
ED: Endothelial dysfunction
ELISA: Enzyme linked immunosorbent assay
eNOS: Endothelial NO synthase
HE: Hematoxylin and eosin
HUVECs: Human umbilical vascular endothelial cells
HW/BW: Heart weight/body weight
IL-10: Interleukin-10
IL-1β: Interleukin-1β
*L*-NAME: *N*^ω^-Nitro-*L*-arginine methyl ester
LVEDP: Left ventricular end-diastolic pressures
LVSP: Left ventricular systolic pressures
MDA: Malondialdehyde
NO: Nitric oxide
PVCA: Perivascular circumferential collagen area
ROS: Reactive oxygen species
SOD: Superoxide dismutase
TAA: Total aorta area
TCM: traditional Chinese medicine
TNF-α: Tumor necrosis factor-α
VG: Van Gieson
XJEK: Xin-Ji-Er-Kang
